# Differential expression of podoplanin in metastatic lymph node is associated with extranodal extension in oropharyngeal cancer

**DOI:** 10.1038/s41598-022-07794-0

**Published:** 2022-03-07

**Authors:** Hye Ran Lee, Jin Roh, Ga Young Gu, Ju Ho Lee, Yoo Seob Shin, Jeon Yeob Jang, Chul-Ho Kim

**Affiliations:** 1 Department of Otolaryngology, School of Medicine, Ajou University, Suwon, Republic of Korea; 2 Department of Pathology, School of Medicine, Ajou University, Suwon, Republic of Korea; 3 Department of Biomedical Sciences, Ajou University Graduate School of Medicine, Suwon, Republic of Korea; 4 Department of Molecular Science and Technology, Ajou University, Suwon, Republic of Korea

**Keywords:** Cancer, Medical research

## Abstract

This study aimed to investigate the spatial distribution and clinical significance of podoplanin expression in the metastatic lymph nodes of oropharyngeal squamous cell carcinomas (OPSCCs). The immunohistochemical podoplanin expression in the metastatic lymph nodes was evaluated in the pathologic specimens of 47 consecutive OPSCC patients. Clinicopathologic factors, including podoplanin expression and extranodal extension (ENE) status, were analyzed. Podoplanin was significantly expressed in the perinodal stroma (*p* = 0.001), and the average score of podoplanin was higher (*p* = 0.008) in ENE-positive lymph nodes than ENE-negative lymph nodes, although intranodal podoplanin expression did not differ significantly between the groups. Multivariable analysis revealed perinodal podoplanin expression as an independent marker of ENE in all the patients and the human papilloma virus (HPV)-positive group (*p* = 0.007 and *p* = 0.018, respectively). Podoplanin is differentially expressed in the metastatic lymph nodes in OPSCC, and its expression in perinodal stroma is associated with ENE, suggesting that podoplanin can be used clinically as a diagnostic biomarker.

## Introduction

Oropharyngeal squamous cell carcinoma (OPSCC), which consists of a subgroup of head and neck cancers (HNCs), includes squamous cell cancers arising from the tonsils, base of the tongue, posterior pharyngeal wall, and soft palate^1,2^. Human papilloma virus (HPV) infection has emerged as a major etiologic factor in the carcinogenesis of OPSCC, accounting for more than 70% of OPSCC cases and contributing to the increasing prevalence of OPSCCs in developed countries^2,3^. HPV-related OPSCC has distinct features and a favorable prognosis compared with non-HPV OPSCC, and these factors have provided the basis for evaluating de-escalation therapy in clinical trials^4^. However, there have been several cases of more aggressive HPV-related OPSCCs, and extranodal extension (ENE) was suggested as a significant prognostic factor for worse outcomes in both HPV and non-HPV OPSCCs^3,5^. ENE, a common finding in cervical nodal metastases of HNCs wherein carcinomatous tissue grows beyond the metastatic lymph node capsule, is a significant determinant of postoperative adjuvant chemoradiation^6^. Histopathological examination of the surgical specimen is performed for the final diagnosis of ENE in the metastatic node. However, there is no standard consensus regarding the precise pathological definition of ENE, and discrepancies in the accuracy of ENE diagnosis range from 21 to 85%^7^.

Podoplanin is a small mucin-type transmembrane glycoprotein that is commonly expressed at the tumor-invasive front; it promotes local invasion and metastasis through the regulation of tumor cell migration and epithelial–mesenchymal transition (EMT)^8,9^. In addition, podoplanin is specifically expressed in the lymphatic system and is a well-known marker for lymphangiogenesis and lymphatic spread of cancer^10^. Elevated podoplanin levels have been reported in various primary tumors, including colorectal cancer, lung squamous carcinoma, and mesothelioma^8,11^. Tissue analysis in 14 types of cancer suggested that podoplanin expression in the tumor stroma is an indication of cancer spread and that expression of podoplanin in cancer cells indicates strong tumor aggressiveness^11^. Recent studies have shown that podoplanin expression in oral squamous cell carcinoma and OPSCC is associated with a high histopathological grade and advanced clinical stage with early nodal metastasis^9,12^. Most studies on podoplanin have considerably focused on primary tumors, and investigations on podoplanin expression in metastatic nodes are limited. A study of patients with oral cancer showed that the relevance of the intratumoral expression of podoplanin with ENE of metastatic nodes presented a similar pattern to that of the invasive front of primary tumors^13^. However, no studies have confirmed the relationship between podoplanin and ENE of OPSCC or the manifestation of podoplanin at the ENE site.

Therefore, this study aimed to comprehensively explore the expression of podoplanin in all areas of metastatic cervical lymph nodes of OPSCC patients, including not only intranodal metastatic tumor sites but also the perinodal stroma corresponding to the tumor stroma of ENE invasive front, through immunohistochemical staining and pathological evaluation.

## Results

### Clinicopathologic characteristics of the participants

In total, 47 patients were enrolled in this study. The mean (range) age of the study population was 60 (45–82) years. Forty patients were males (85%) and 7 were females (15%); 27 patients (57%) had a history of smoking, and the remaining 20 patients (43%) were non-smokers. The demographic characteristics of these patients had no significant correlation with the ENE status (Table 1). The most common tumor invasion location in the oropharyngeal subsite was the palatine tonsil in 39 cases (83%) and the base of the tongue in 8 cases (17%). The ENE status was not significantly associated with the tumor subsite or any other primary tumor pathologic features, such as the T classification, tumor grade, lymphovascular invasion (LVI), and perineural invasion (PNI). In the ENE-positive group, 21 patients (75%) received adjuvant concurrent chemoradiation therapy (CCRT); in the ENE-negative group, 6 patients (32%) received adjuvant CCRT; the remaining 13 patients (68%) received adjuvant radiation therapy, which represented statistical significance as shown in Table 1.

**Table 1:** Clinicopathological characteristics of the patients in relation to ENE status (N = 47).

	Total (N = 47)	ENE-negative (N = 19)	ENE-positive (N = 28)	*P* value^a^
**Age (years)**				0.525
Median, [25-75th percentile]	60 [59–62]	59 [57–62]	61 [59–63]	
**Gender, N (%)**				0.102
Male	40 (85)	14 (74)	26 (93)	
Female	7 (15)	5 (26)	2 (7)	
**Smoking history, N (%)**				0.764
Smoker	27 (57)	10 (53)	17 (61)	
Non-smoker	20 (43)	9 (47)	11 (39)	
**Tumor subsite, N (%)**				0.445
Palatine tonsil	39 (83)	17 (90)	22 (79)	
Base of tongue	8 (17)	2 (10)	6 (21)	
**pT classification, N (%)**				0.111
T1 – T2	33 (70)	16 (84)	17 (61)	
T3 – T4	14 (30)	3 (16)	11 (39)	
**Tumor grade, N (%)**				0.945
Well differentiated	1 (2)	0 (0)	1 (4)	
Moderately differentiated	24 (51)	9 (47)	15 (54)	
Poorly differentiated	17 (36)	8 (42)	9 (32)	
Unknown	5 (11)	2 (11)	3 (11)	
**Lymphovascular invasion, N (%)**				0.087
Absent	16 (34)	10 (53)	6 (21)	
Present	22 (47)	7 (37)	15 (54)	
Unknown	9 (19)	2 (10)	7 (25)	
**Perineural invasion, N (%)**				0.464
Absent	33 (70)	15 (79)	18 (64)	
Present	5 (11)	2 (11)	3 (11)	
Unknown	9 (19)	2 (11)	7 (25)	
**Metastatic lymph node, N (%)**				
**Maximum of diameter (cm)**				0.072
≤ 3.2 (lower half of median)	26 (55)	14 (74)	12 (43)	
> 3.2 (upper half of median)	21 (45)	5 (26)	16 (57)	
**Number**				0.310
≤ 4	35 (75)	16 (84)	19 (68)	
> 4	12 (25)	3 (16)	9 (32)	
**Metastasis pattern, N (%)**				0.767
Solid	25 (53)	11 (58)	14 (50)	
Cystic	22 (47)	8 (42)	14 (50)	
**Podoplanin expression, N (%)**				
Intranodal				1.000
Positive	11 (23)	4 (21)	7 (25)	
Negative	36 (77)	15 (79)	21 (75)	
**Perinodal stroma**				**0.001**
Positive	35 (75)	9 (47)	26 (93)	
Negative	12 (25)	10 (53)	2 (7)	
**HPV status, N (%)**				0.720
Positive	38 (81)	16 (84)	22 (79)	
Negative	9 (19)	3 (16)	6 (21)	
**Treatment, N (%)**				**0.002**
Surgery only	1 (2)	0 (0)	1 (4)	
Adjuvant RT	19 (40)	13 (68)	6 (21)	
Adjuvant CCRT	27 (57)	6 (32)	21 (75)	

*RT* radiation therapy, *CCRT* concurrent chemoradiation therapy, *SD* standard deviation.

Significant values are in bold.

*P* value^a^: statistical analyses between ENE-positive versus ENE-negative of metastatic node.

### Podoplanin expression in metastatic lymph nodes

**Figure 1:**
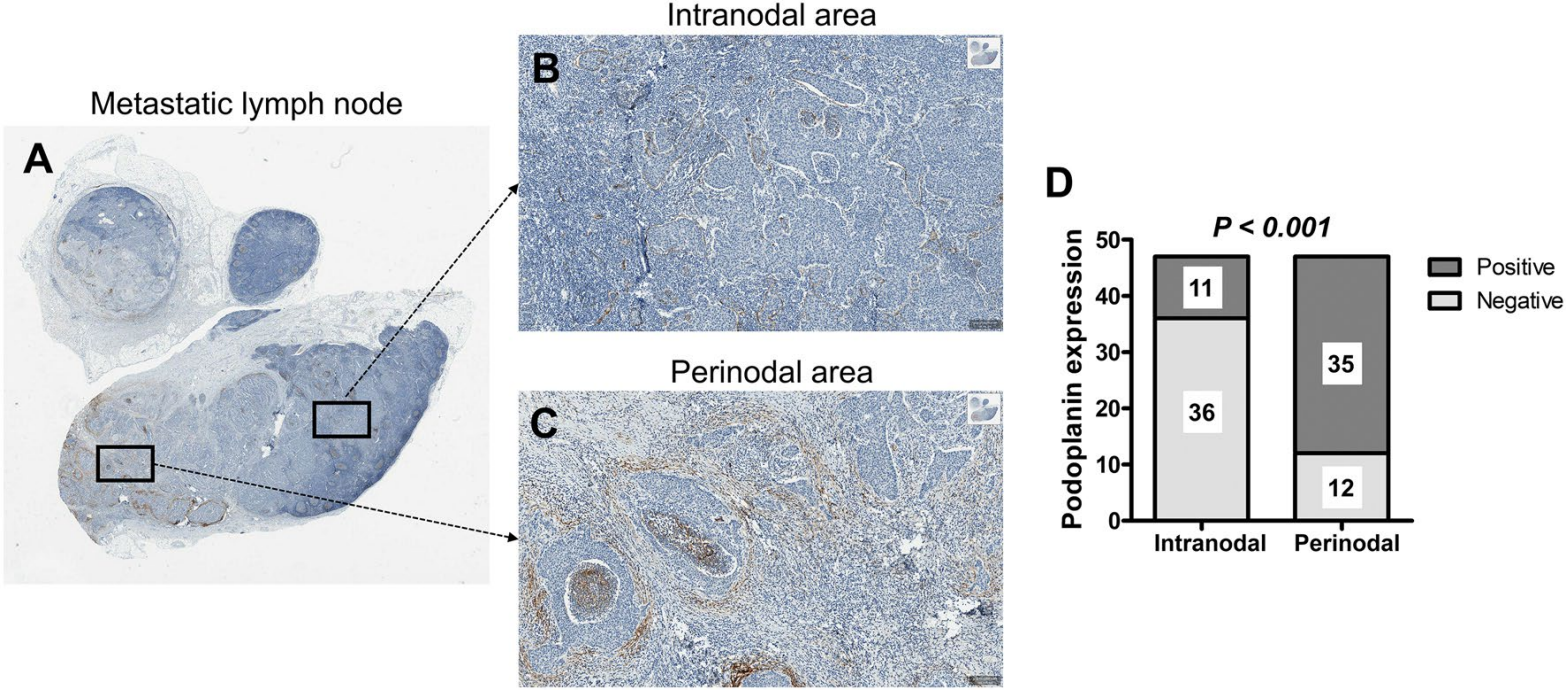
Representative histopathological images of podoplanin (PDPN) expression in the whole area of a metastatic lymph node. (**A**) Comparative representative images of PDPN expression between (**B**) intranodal and (**C**) perinodal area in one metastatic lymph node (both indicated with black squares). (**B**) Scant PDPN expression was observed in the overall intranodal metastatic area. (**C**) Prominent stromal PDPN expression was noted in the perinodal area. (**D**) Proportion of patients positive for PDPN expression was compared in each of the intranodal and perinodal areas. There were significantly more patients of PDPN-positive in the perinodal area of metastatic lymph node. (*P* < 0.001). Original magnification: (**A**) × 10, (**B**) × 200, and (**C**) × 200.

**Figure 2:**
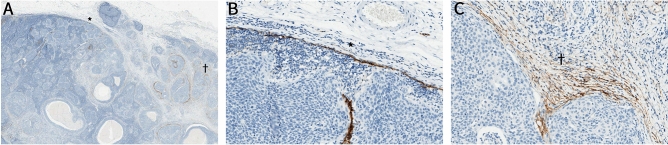
Representative histopathological image of podoplanin (PDPN) expression in perinodal stromal cells based on the presence of extranodal extension (ENE). (**A**) Comparison of PDPN expression in perinodal stromal cells between the area with ENE (indicated by a dagger [^†^]) and the area without ENE (indicated by an asterisk [*]) in a metastatic lymph node. (**B**) In the area without ENE (*), there is no PDPN expression in the perinodal stromal cells. PDPN expression in subcapsular and intranodal lymphatics is considered as an internal control. (**C**) In the area with ENE (^†^), moderate-intensity PDPN expression is observed. Original magnification: (**A**) × 10, (**B**) × 200, and (**C**) × 200.

**Figure 3:**
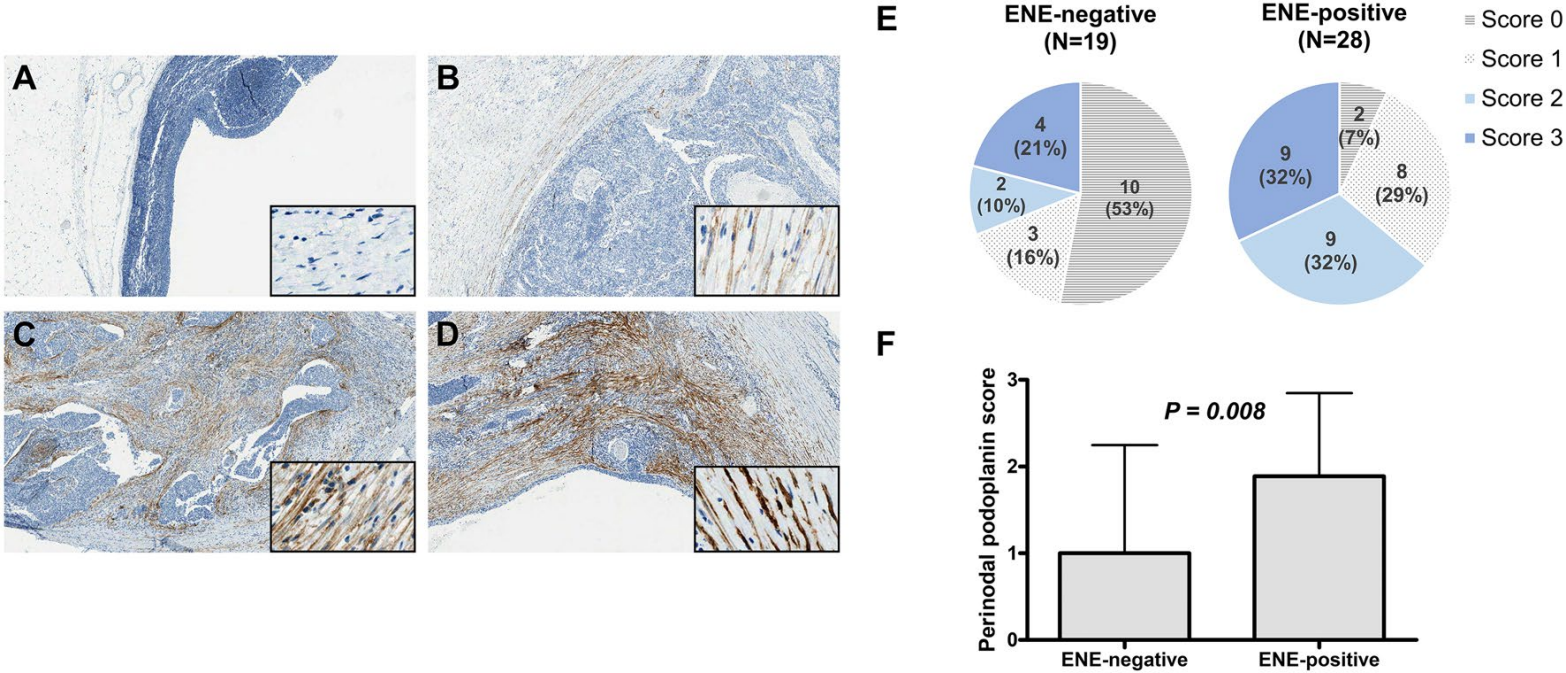
Representative images of podoplanin (PDPN) expression in the cytoplasm of perinodal stromal cells. (**A**) A PDPN score of 0 indicates the absence of PDPN expression in perinodal stromal cells. (**B**–**D**) In PDPN-positive cases, more than 30% of perinodal stromal cells show PDPN expression. (B) A PDPN score of 1 indicates weak PDPN expression in stromal cells that are adjacent to extranodal infiltrating tumor cells; (**C**) A PDPN score of 2 shows moderate PDPN expression; and (**D**) a PDPN score of 3 shows strong PDPN expression in stromal cells that are adjacent to extranodal infiltrating tumor cells. Original magnification: × 40 (inset, × 400). (**E**) Pie charts showing the distribution of patients according to PDPN score in each of the ENE-negative (left) and ENE-positive (right) groups. (**F**) Bar chart comparing the average score of perinodal PDPN expression between the ENE-negative and positive groups (1.00 ± 1.247 vs. 1.89 ± 0.956; *p* = 0.008).

The mean (range) metastatic node size (maximum axial diameter) was 3.2 (0.9–8.5) cm. The size and number of metastatic nodes and the metastasis pattern of solid or cystic lesions were unrelated to the occurrence of ENE. Podoplanin expression in the intranodal metastatic tumor area was detected in only 11 (23%) of the total participants and showed no association with the ENE status (Fig. 1). Podoplanin expression in the perinodal stroma of metastatic nodes was detected in 35 patients (75%), and a significant association was identified in the ENE-positive group (93%; *p* = 0.001) compared with the ENE-negative group (47%). Figure 2 demonstrates a histopathological image of podoplanin expression in the perinodal stroma, which was limited to the area with ENE in one metastatic node compared with the region without ENE. Moreover, the distribution of patients with a high podoplanin expression score of 2 or 3 in the perinodal stroma was much higher in the ENE-positive group than in the ENE-negative group (Fig. 3, 64% vs. 31%); the average score of podoplanin expression in the perinodal stroma was significantly higher in the ENE-positive group than in the ENE-negative group (Fig. 3, 1.00 ± 1.247 vs. 1.89 ± 0.956; *p* = 0.008). In ENE-positive group, the amount of ENE was confirmed that microscopic ENE in 10 patients and major ENE in 18 patients out of a total of 28 patients. Intranodal (20% vs 28%) and perinodal stroma (80% vs 100%) podoplanin expression were detected more in major ENE than in microscopic ENE, although there was no statistical significance. There was also no significant difference between the podoplanin scores of ENE subdivisions in the intranodal tumor area and perinodal stroma. In both microscopic- and major-ENE, podoplanin expression and mean score were significantly higher in perinodal stroma than in intranodal metastatic tumor area (0.30 ± 0.675 vs. 2.00 ± 1.247; *p* = 0.001, 0.56 ± 1.042 vs. 1.83 ± 0.786, *p* < 0.001) (Supplementary table 1). Among all the patients, 38 patients (81%) were classified as the HPV-positive group; the remaining 9 patients (19%) constituted the HPV-negative group, which had no significant correlation with ENE (Table 1).

### Correlation of podoplanin expression with HPV and ENE status

Table 2 presents the details of podoplanin expression in the perinodal stroma in the HPV-positive and HPV-negative groups, along with their correlation with the ENE status. In the HPV-positive group, podoplanin expression in perinodal stroma was identified significantly in 95% of ENE-positive cases (*p* = 0.002). Moreover, the mean podoplanin score was significantly higher in the ENE-positive group than in the ENE-negative group (2.05 ± 0.899 vs. 1.00 ± 1.211; *p* = 0.004). There was no significant difference in podoplanin expression according to the ENE status in the HPV-negative group.

**Table 2:** Comparison of podoplanin expression on perinodal stroma according to HPV and ENE status (N = 47).

Group	ENE-negative (N = 19)	ENE-positive (N = 28)	*P* value^a^
**HPV-positive (N = 38)**			
**Podoplanin expression, N (%)**			**0.002**
Positive	8 (50)	21 (95)	
Negative	8 (50)	1 (5)	
**Podoplanin score, N (%)**			**0.004**
0	8 (50)	1 (5)	
1	3 (19)	5 (23)	
2	2 (12)	8 (36)	
3	3 (19)	8 (36)	
Mean (SD)	1.00 (1.211)	2.05 (0.899)	
**HPV-negative (N = 9)**			
**Podoplanin expression, N (%)**			0.464
Positive	1 (33)	5 (83)	
Negative	2 (67)	1 (17)	
**Podoplanin score, N (%)**			0.722
0	2 (67)	1 (17)	
1	0 (0)	3 (50)	
2	0 (0)	1 (17)	
3	1 (33)	1 (17)	
Mean (SD)	1.00 (1.732)	1.33 (1.033)	

SD: Standard deviation.

Significant values are in bold.

*P* value^a^: statistical analyses between ENE-positive versus ENE-negative of metastatic node.

### Predictors of ENE including podoplanin expression of perinodal stroma in metastatic lymph nodes

Table 3 displays the results of the univariate and multivariable analyses in the entire study cohort and the HPV-positive group, and they determined the impact of risk factors presumed to be related to ENE. The univariate analysis of the study population showed that a larger maximum size of metastatic node (*p* = 0.024), the presence of perinodal stromal podoplanin expression (*p* = 0.002), and a higher score of podoplanin expression (*p* = 0.031) were significantly associated with ENE. In the multivariable analysis, the podoplanin expression in the perinodal stroma was significantly associated with ENE (*p* = 0.007), while the maximum size of the metastatic node did not reach statistical significance (*p* = 0.073). Univariate analysis in the HPV-positive group demonstrated that lymphovascular invasion was a significant risk factor for ENE (*p* = 0.048) in addition to the presence of perinodal stromal podoplanin expression (*p* = 0.008) and a high score of the expression (*p* = 0.014). However, only the podoplanin expression in the perinodal stroma was a statistically significant risk factor in the multivariable analysis of the HPV-positive group (*p* = 0.018).

**Table 3:** Binomial logistic regression analysis for predicting risk factors of ENE.

	Univariate model			Multivariable model		
	HR	95% CI	*p*	HR	95% CI	*p*
***Whole patient (N* = *47)***						
**Tumor subsite**						
Palatine tonsil	1 (Ref.)					
Base of tongue	2.318	0.415–12.958	0.338			
**T stage**						
T1-2	1 (Ref.)					
T3-4	3.451	0.811–14.678	0.094			
**Tumor grade**						
WD/MD	1 (Ref.)					
PD	1.185	0.166–8.471	0.866			
**Lymphovascular invasion**						
Absent	1 (Ref.)					
Present	3.571	0.924–13.811	0.065			
**Perineural invasion**						
Absent	1 (Ref.)					
Present	1.250	0.184–8.491	0.819			
**Maximum size of metastatic node (cm)**						
≤ 3.2	1 (Ref.)			1 (Ref.)		
> 3.2	4.327	1.213–15.439	**0.024**	4.329	0.873–21.462	0.073
**Number of metastatic nodes**						
≤ 4	1 (Ref.)					
> 4	2.526	0.583–10.945	0.215			
**Metastasis pattern**						
Cystic	1 (Ref.)					
Solid	0.727	0.225–2.353	0.595			
**Podoplanin expression (intranodal)**						
Absent	1 (Ref.)					
Present	1.250	0.310–5.048	0.754			
**Podoplanin expression (perinodal stroma)**						
Absent	1 (Ref.)			1 (Ref.)		
Present	14.444	2.647–78.823	**0.002**	12.587	2.004–79.074	**0.007**
**Podoplanin score (perinodal stroma)**						
0–1	1 (Ref.)					
2–3	3.900	1.131–13.454	**0.031**			
**HPV status**						
Negative	1 (Ref.)					
Positive	0.688	0.149–3.169	0.631			
***HPV-positive group (N* = *38)***						
**Tumor subsite**						
Palatine tonsil	1 (Ref.)					
Base of tongue	1.556	0.248–9.750	0.637			
**T stage**						
T1-2	1 (Ref.)					
T3-4	2.625	0.454–15.162	0.281			
**Tumor grade**						
WD/MD	1 (Ref.)					
PD	0.500	0.125–1.999	0.327			
**Lymphovascular invasion**						
Absent	1 (Ref.)					
Present	4.950	1.017–24.095	**0.048**			
**Perineural invasion**						
Absent	1 (Ref.)					
Present	0.429	0.034–5.333	0.510			
**Maximum size of metastatic node (cm)**						
≤ 3.2	1 (Ref.)			1 (Ref.)		
> 3.2	4.333	0.959–19.579	0.057	3.618	0.533–24.582	0.188
**Number of metastatic nodes**						
≤ 4	1 (Ref.)					
> 4	3.267	0.578–18.464	0.180			
**Metastasis pattern**						
Cystic	1 (Ref.)					
Solid	0.500	0.134–1.862	0.301			
**Podoplanin expression (intranodal)**						
Absent	1 (Ref.)					
Present	0.684	0.119–3.933	0.671			
**Podoplanin expression (perinodal stroma)**						
Absent	1 (Ref.)			1 (Ref.)		
Present	21.000	2.252–195.816	**0.008**	17.128	1.629–180.114	**0.018**
**Podoplanin score (perinodal stroma)**						
0–1	1 (Ref.)					
2–3	5.867	1.427–24.113	**0.014**			

Significant values are in bold.

*HR* hazard ration, *CI* confidence interval, *WD* well differentiated, *MD* moderately differentiated, *PD* poorly differentiated.

## Discussion

ENE is a predictor of local recurrence and distant metastasis in HNC. Accordingly, postoperative adjuvant chemoradiation treatment is commonly recommended^14^. In HPV-related OPSCC, ENE has been excluded from the latest AJCC N-staging determinants, as it exclusively showed a better treatment response and prognosis. However, the mechanisms mediating this difference in prognosis have not yet been clearly identified, and the association between ENE and worse outcome has been reported in HPV-positive OPSCC^15,16^. In our study cohort, adjuvant treatment was administered to most of the patients with ENE. Clinical trials for de-intensified treatment for HPV-positive OPSCC are being conducted; however, an unequivocal de-escalation protocol has not yet been established with regard to the presence or absence of ENE^17^.

In this study, the results of the multivariable analysis showed that podoplanin expression in the perinodal stroma of metastatic lymph nodes in OPSCC patients is a significant factor associated independently with the presence of ENE. Mermod et al. proposed that podoplanin expression in the intratumoral area in the metastatic node was an independent factor associated with ENE in oral cavity cancers^13^. This is the first published research evaluating the expression of podoplanin in the metastatic node and has considerable value. However, only the expression level of podoplanin in the metastatic node was presented as a simple quantification, and the expression difference by region in the lymph node was not precisely evaluated. In addition, while their target was oral cancer, we conducted a study on oropharyngeal cancer. Therefore, in this study, we analyzed whether there was a heterogeneity in podoplanin expression in each subarea of metastatic node in OPSCC, which has recently emphasized the necessity to understand the pathophysiology of ENE different from other HNCs. In our study, podoplanin expression in the intranodal metastatic tumor area did not show a considerable effect on ENE, regardless of the HPV status, at least in OPSCC. Regarding podoplanin as a biomarker for tumor cell migration, our findings suggest a more meaningful association between ENE and podoplanin activity, especially in terms of the invasive front. Besides, the marked presence of stromal podoplanin in ENE is considered to represent the ENE of cancer with desmoplastic reaction around perinodal infiltrative edge in the metastatic node^11^. This has the meaning of emphasizing the importance of the microenvironment in cancer infiltrative edge where ENE occurs, which requires further elucidation by additional research.

As ENE is a representative aggressive feature for various cancers, including HNC, efforts to identify clinicopathologic features that can predict ENE have been undertaken; however, there is no consensus on factors related to the primary tumor, and the controversy of ENE diagnosis itself persists^18^. In the absence of an evident clinical manifestation, except for radiological findings that could facilitate a definitive preoperative diagnosis of ENE, molecular mechanisms and biomarkers that may induce ENE have been explored^7,18^. We subdivided the entire ENE-positive group into microscopic ENE and major ENE based on the amount of ENE beyond the metastatic node capsule of 2 mm^19^. In our results, there were no significant differences in podoplanin expression and podoplanin score by subdivision according to ENE amount in both region of metastatic node. Meanwhile, in both ENE subdivisions, podoplanin expression and mean score were significantly higher in the perinodal stroma than in the intranodal area, this seems to be the result of reproducing our main data in which podoplanin was significantly expressed in perinodal stroma in the entire ENE-positive group. Even if major ENE showed worse prognosis than microscopic ENE in several studies of non-OPSCC ENE, the universal consensus was not achieved, and especially in HPV-related OPSCC, the value of ENE subdivision is more ambiguous^20^. Therefore, verification of the relationship between podoplanin expression which reflects tumor aggressiveness, and ENE subcategories could be contributed to establish the clinical implication of ENE progression. For this, it seems that analysis after accumulating further data is necessary.

In addition, the tumor microenvironment (TME) has recently garnered increased focus because of its potential to affect the behavior of cancer cells; the significance of biomarkers related to the TME is increasing^21^. Cancer-associated fibroblasts (CAFs), whose proliferation and secretion of growth factors are abnormally elevated owing to cancer, are the main constituent cells of the TME^22^. Podoplanin expression in CAFs was recently proposed as a new biomarker and has been identified in various malignancies, including breast and lung cancers, and there is evidence of the involvement of TME in ENE development in the nodal microenvironment of oral cancers^21,22^. CAFs are involved in tumor progression in cancers including HNC, through lymphangiogenesis and EMT, similar to the main mechanism of action of podoplanin^11,23^. In the present study, CAFs were assumed to be the major contributory cell to podoplanin expression in the perinodal stroma. Our preliminary immunohistochemistry analysis of several metastatic nodes showed the podoplanin and smooth muscle actin protein (SMA) had the same distribution of expression in perinodal stroma (Supplementary Fig. S1). However, molecular-level studies of CAF-related mechanisms of generating ENE in OPSCC, including co-staining with α-SMA (a characteristic marker of CAF)^22^ and the effect of HPV infection, should be conducted to validate the results of this study.

This study had a few limitations. The results showing a significantly prominent podoplanin expression on perinodal stroma in the HPV-positive group could be a consequence of the small sample size of the HPV-negative group, which makes it difficult to presume that this result represents the occurrence of ENE by an HPV-specific mechanism. Therefore, comparative large-scale multicenter studies including HPV-negative patients are needed to clarify this association.

In conclusion, this study confirmed that regional heterogeneity of podoplanin expression in metastatic lymph nodes for the first time. It also revealed a novel finding that increased podoplanin expression in the perinodal stroma of the metastatic lymph node is strongly associated with and an independent predictor of ENE in OPSCC. The application of podoplanin as a diagnostic tool in clinical practice is based on additional research that can conclusively confirm a relationship between ENE and podoplanin; thus, studies on the mechanisms of ENE development in the lymph node microenvironment to identify independent prognostic markers of ENE in OPSCC are warranted.

## Methods

### Study participants

The study participants were identified from a retrospective chart review of medical records of patients treated at the department of head and neck surgery of our hospital from February 2013 to January 2020. The inclusion criteria were pathologically proven squamous cell carcinomas of the oropharynx involving the palatine tonsil and base of the tongue, presence of cervical lymph node metastasis, and patients who underwent surgical resection as the initial treatment. Patients who underwent salvage surgery, those with distant metastases on initial presentation, and those diagnosed as having no lymphatic metastasis on final histopathology were excluded. The study protocol was approved by the Institutional Review Board of Ajou University Hospital (approval No. BMR-KSP-20-156) and was allowed to waive the requirement to obtain informed consent because of the retrospective nature of this study. The metastatic cervical lymph node specimens of 47 consecutive patients diagnosed with OPSCC were analyzed. Cancer staging was performed according to the 2017 American Joint Committee on Cancer (AJCC) staging criteria, 8th edition^24^. The high-risk HPV status of each tumor was assessed using either p16 immunohistochemical staining or the Cobas® HPV test (Roche Molecular Diagnostics, Mannheim, DE, Germany) in accordance with the manufacturer’s instructions.

### Immunohistochemical and pathologic evaluations

ENE was defined as spread of carcinoma cells outside the lymph node capsule with infiltration of perinodal soft tissue^19^. The amount of ENE was additionally subdivided in two categories. The “microscopic ENE” was defined as the presence of ENE ≤ 2 mm beyond the lymph node capsule and the “major ENE” was defined as the presence of ENE > 2 mm beyond the lymph node capsule. H&E stained slides were reviewed by one board certified head and neck pathologist (JR) in a blind manner. Immunohistochemical staining for podoplanin and SMA was performed using the Bench Mark XT (Ventana Medical Systems, Oro Valley, AZ, USA) according to the manufacturer’s instructions. Briefly, 4-μm-thick sections were deparaffinized in xylene and dehydrated in graded ethanol solutions. Antigen retrieval with the cell conditioning buffer 1 (CC1) was performed for 40 min. The sections were incubated with the primary antibodies, podoplanin (D2-40; 322 M, 1:20, Cell Marque, Rocklin, CA, USA) and SMA (1A4, 1:100, Cell Marque, Rocklin, CA, USA), for 1 h and with the secondary anti-HRP antibody for 32 min. Counterstaining was performed with hematoxylin II for 10 min and subsequent bluing for 6 min. The whole-tissue section slides were examined under a light microscope. The presence of fibroblasts around the metastatic lymph nodes was evaluated by a pathologist (JR) who was blinded to the clinical data. The cytoplasmic podoplanin expression was semi-quantitatively evaluated. Cases with podoplanin expression in > 30% of the fibroblasts around the metastatic lymph nodes were categorized as positive; all others were classified as negative. Positive cases were further graded from 1 to 3, according to the podoplanin protein expression intensity (1, weak expression; 2, moderate expression; and 3, strong expression).

### Statistical analysis

To compare the clinical parameters between the ENE-positive and ENE-negative groups, the chi-square or Fisher’s exact test was conducted for categorical variables and the independent *t*- or Mann–Whitney *U* test was conducted for continuous variables. Univariate and multivariable model analyses were performed using the binomial logistic regression test to determine the effect of each clinicopathologic feature of tumors and podoplanin expression as risk factors for the occurrence of ENE. SPSS Version 18 (SPSS, Inc., Chicago, IL, USA) was used for the analyses. A *p*-value of < 0.05 was considered statistically significant.

### Ethical Statement

The study protocol was approved by the Institutional Review Board of Ajou University Hospital (approval No. BMR-KSP-20-156) and was allowed to waive the requirement to obtain informed consent. All methods were performed in accordance with relevant guidelines and regulations.

### Consent to participate

The Requirement of informed consent was waived because of the retrospective nature of this study.

## Supplementary Information

Supplementary Information.
