# Supplementary material for: Differential expression of podoplanin in metastatic lymph node is associated with extranodal extension in oropharyngeal cancer
Source: Sci Rep. 2022 Mar 7;12:3665. doi: 10.1038/s41598-022-07794-0 (PMC8901644; doi:10.1038/s41598-022-07794-0)

Supplementary materials

**Title: Differential expression of podoplanin in metastatic lymph node is associated with extranodal extension in oropharyngeal cancer**

*Hye Ran Lee^1†^, Jin Roh, MD^2†^, Ga Young Gu^1^, Ju Ho Lee^1^, Yoo Seob Shin^1^, Jeon Yeob Jang^1,3*^, Chul-Ho Kim^1,4*^*

**Table S1.** Comparison of podoplanin expression on intranodal and perinodal stroma according to amount of ENE (N=28)

| ENE-positive  (N=28) | Intranodal  podoplanin expression  (N=7) | Perinodal stroma podoplanin expression  (N=26) | *P-value^a^* | | |
| --- | --- | --- | --- | --- | --- |
| *Presence of podoplanin expression, N (%)* | | | | |  |
| Microscopic ENE  (N=10) | 2 (20) | 8 (80) | | **0.005** |  |
| Major ENE  (N=18) | 5 (28) | 18 (100) | | **< 0.001** |  |
| *P-value^a^* | 1.000 | 0.119 | |  |  |
| *Podoplanin score, Mean (SD)* | | | | | |
| Microscopic ENE  (N=10) | 0.30 (0.675) | 2.00 (1.247) | **0.001** | | |
| Major ENE  (N=18) | 0.56 (1.042) | 1.83 (0.786) | **< 0.001** | | |
| *P-value^a^* | 0.493 | 0.667 |  | | |

SD: Standard deviation

*P-value^a^*: statistical analyses between microscopic ENE versus major ENE of ENE-positive node

**Figure S1.** Immunohistochemistry analysis represented that both D2-40 (podoplanin) and smooth muscle actin (SMA) had the same distribution of expression within the metastatic nodes.


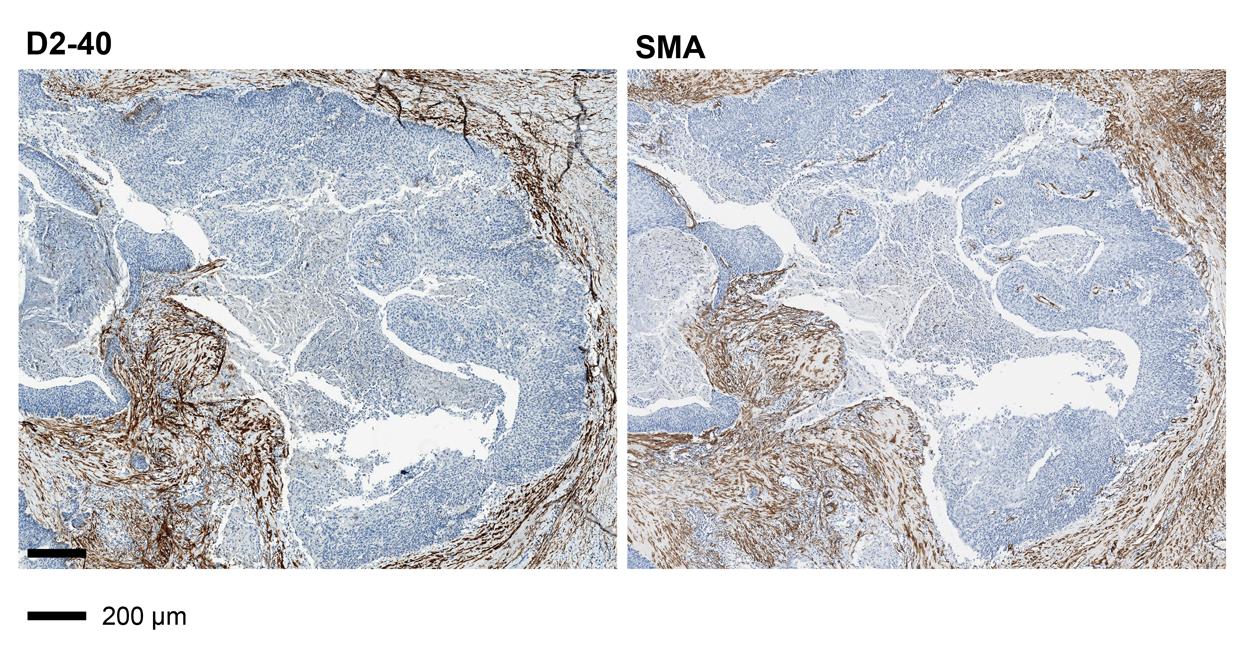

Supplement: Supplementary file 1 — Supplementary Information. [file 41598_2022_7794_MOESM1_ESM.docx]
